# Detection, Characterization, and Clinical Application of Mesenchymal Stem Cells in Periodontal Ligament Tissue

**DOI:** 10.1155/2018/5450768

**Published:** 2018-08-26

**Authors:** Atsushi Tomokiyo, Shinichiro Yoshida, Sayuri Hamano, Daigaku Hasegawa, Hideki Sugii, Hidefumi Maeda

**Affiliations:** ^1^Division of Endodontics, Kyushu University Hospital, Fukuoka, Japan; ^2^Department of Endodontology and Operative Dentistry, Faculty of Dental Science, Kyushu University, Fukuoka, Japan; ^3^OBT Research Center, Kyushu University, Fukuoka, Japan

## Abstract

Mesenchymal stem cells (MSCs) are a kind of somatic stem cells that exert a potential to differentiate into multiple cell types and undergo robust clonal self-renewal; therefore, they are considered as a highly promising stem cell population for tissue engineering. MSCs are identified in various adult organs including dental tissues. Periodontal ligament (PDL) is a highly specialized connective tissue that surrounds the tooth root. PDL also contains MSC population, and many researchers have isolated them and performed their detailed characterization. Here, we review the current understanding of the features and functions of MSC population in PDL tissues and discuss their possibility for the application of PDL regeneration.

## 1. Introduction

**Figure 1:**
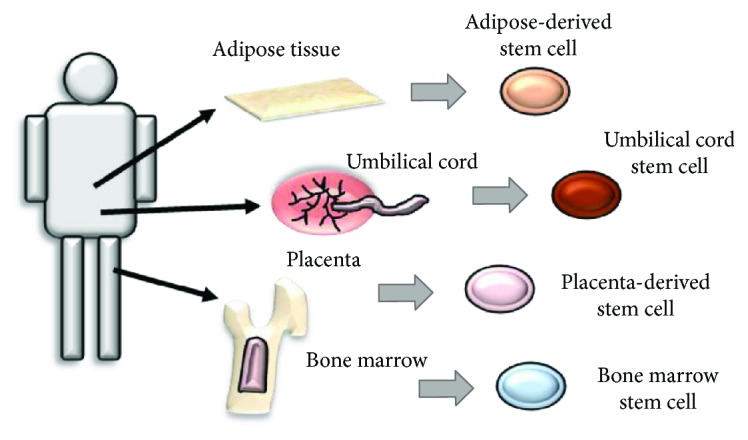
Origins of MSCs. MSCs are isolated from several somatic tissues such as bone marrow, adipose tissue, umbilical cord, and placenta. They have different biological characters and potential to be used as clinical options in regenerative medicine.

Mesenchymal stem cells (MSCs) have been reported to be isolated from a variety of tissues such as bone marrow, adipose tissue, umbilical cord, and placenta [1, 2]. They show the lineage-specific characters and possess the capacity to regenerate tissues that they are included (Figure 1). Because of their accessibility, high growth capacity, and multipotency [3], cell-based medicine utilizing MSCs is expected to facilitate tissue regeneration in various clinical applications. Recently, MSC-like populations have also been identified in dental tissues [4, 5]. These postnatal progenitors have MSC qualities, including the capacity for self-renewal and multilineage differentiation potential. Therefore, they will give rise to candidates in regenerative medicine for not only dental tissues but also other somatic tissues.

In 1981, establishment of embryonic stem (ES) cells from the inner cell mass of mouse blastocysts [6] and human ES cells has also derived from human blastocysts [7]. Human ES cells possess unrestricted proliferative ability despite its multipotency, but ethical issues always follow. In addition, researchers also face the problem of intense immunorejection in case of transplantation of cells differentiated from ES cells. Therefore, regenerative medicine using human ES cells has not yet been achieved. However, large amounts of experiment have been carried out to overcome these problems. Generation of induced pluripotent stem (iPS) cells from adult human dermal fibroblasts with lentiviral transfection of four genes has reported for the first time in the world [8]. Human iPS cells can overcome problems that ES cells contain because iPS cells can be generated from the patients themselves, so these cells have been attracting great attention in the scientific field of stem cell biology. However, because of the risks of malignancy and teratoma formation of transplanted iPS cells, it is also hard for iPS cells to be a first choice for regenerative medicine. Therefore, the major device of clinical application in recent cell transplantation therapy is MSCs derived from somatic tissues.

PDL is a highly specialized connective tissue that surrounds the tooth root, connects it with the tooth socket bone, and involves proper tooth homeostasis, repair, and nutrition [9]. PDL tissue originates from the dental follicle that surrounds the developing tooth germs during the early stages of tooth development. Mammalian teeth develop from sequential and reciprocal interactions between the oral epithelium and the cranial neural crest-derived mesenchyme, and the dental follicle is an ecto-mesenchymal-derived component of the tooth germs. Interestingly, PDL cells exhibited a closer match to dental follicle cells in global gene expression profiles than did alveolar bone osteoblasts and cementoblasts [10]. Therefore, the PDL tissue mainly consisted of dental follicle-derived mesenchymal cells. The PDL cell population has been considered to include progenitor cells that migrate from locations close to blood vessels and endosteal spaces [11]. After that, Seo et al. first reported the existence of the MSC population in PDL tissue (PDL-MSCs) [5] and many researchers succeeded to isolate PDL-MSCs from PDL tissue. The aim of this article is to summarize the status in PDL-MSCs, the potential benefits of using PDL-MSCs to treat damaged PDL tissues, and future prospectives of PDL-MSC-based regenerative periodontal therapies.

### 1.1. Multipotency of PDL-MSCs

**Figure 2:**
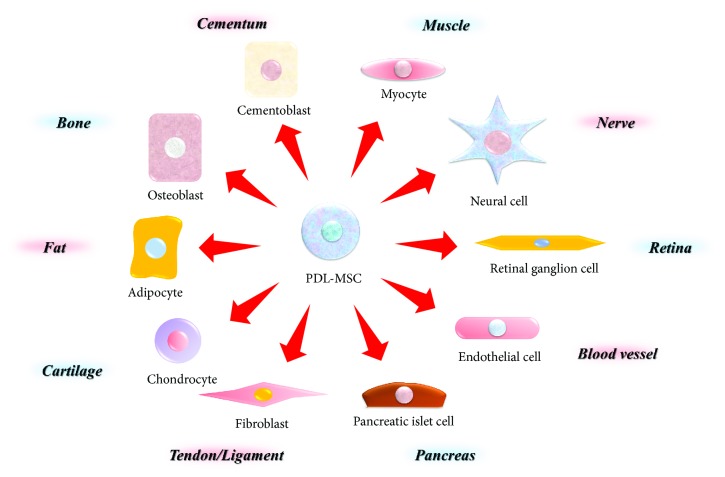
Multipotency of PDL-MSCs. PDL-MSCs are highly multipotent stem cells because they have the capacity to differentiate into osteoblasts, adipocytes, chondrocytes, fibroblast (tenocytes), pancreatic islet cells, endothelial cells, retinal ganglion cells, neural cells, myocytes, and cementoblasts.

PDL-MSCs have the ability to differentiate into several cells under defined culture conditions (Figure 2). A previous study revealed that PDL-MSCs differentiate into osteoblast/cementoblast-like cells and adipocytes [5]. Another report showed that PDL-MSCs had the ability to differentiate into osteogenic, adipogenic, and chondrogenic cells [12]. Recently, it was reported that PDL-MSCs have the potential for neurogenic and angiogenic differentiation. PDL-derived spheres contained MSC-like cells that were capable of differentiating into both neural and mesodermal progeny [13]. In addition, PDL-MSCs could differentiate into Schwann cells via the Erk1/2 signalling pathway [14]. Okubo et al. reported the differentiation of PDL-MSCs into endothelial cells by the activation of the PI3K signaling pathway [15]. Lee et al. generated islet-like cells from PDL-MSCs; the cells expressed endoderm- and pancreas-related genes and secreted insulin in response to high concentrations of glucose [16]. PDL-MSCs also differentiated into retinal ganglion-like cells that expressed neuronal and retinal markers, formed synapses, and responded with glutamate-induced calcium [17]. Additionally, the short-term mechanical strain could induce PDL-MSCs into cardiac myocytes that expressed cardiac cell markers, sarcomeric actin, and cardiac troponin T proteins [18].

Moreover, PDL-MSCs have the potential for regeneration of several organs. When PDL-MSCs were implanted into a surgically created calvarial critical size defect, a thick layer composed of fibrous connective tissue and newly formed bone covered the defect [19]. Following the transplantation of PDL-MSCs into the *in vivo* tendon defect animal models, the cells could differentiate into tenocytes that expressed tendon markers including Tnmd, Eya1, Eya2, and SCX and induced tendon regeneration [20]. Injected PDL-MSCs into the adult mouse brain survived, migrated, and gave rise to mature neural cells [21]. Interestingly, several grafted cells were present in the stem cell niches, suggesting that PDL-MSCs could differentiate into neural stem cells *in vivo*.

### 1.2. Self-Renewal Capacity of PDL-MSCs

**Figure 3:**
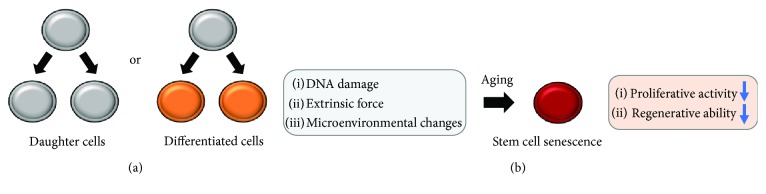
Self-renewal mechanisms of MSCs. (a) Self-renewal of stem cells is divided into two tasks, generation of daughter cells or differentiated cells. (b) Unfortunate accumulation of DNA damage, extrinsic force, and/or the changes of surrounding environment cause senescence and decline in functions of stem cells.

Stem cells are defined by their ability to make new stem cells (self-renewal) [22]. This ability is divided into two types according to how they self-renew. One is generating daughter cells with stem cell fate, and the other is generating differentiated cells (Figure 3(a)). As a result, stem cells could maintain and regenerate tissues throughout the life of each individual [23].

PDL-MSCs also exhibit self-renewal capacity. Surprisingly, PDL-MSCs have reported to possess a higher growth potential than bone marrow-derived MSCs (BM-MSCs); BM-MSCs stopped proliferation about 50 population doublings; however, PDL-MSCs maintained a proliferative capacity beyond 100 population doublings [24]. Mechanical stress is suggested to involve the high proliferative potential of PDL-MSCs because the PDL tissue is continually exposed to mechanical stress caused by mastication or occlusion. A previous report revealed that mechanical loading enhanced PDL-MSC proliferation [25]. Our recent study suggested the mechanism of mechanical loading-induced proliferation in PDL-MSCs; mechanical loading induced the production and secretion of IL-11 in PDL cells and osteoblasts locating around the PDL tissue, and secreted IL-11 increased the self-renewal capacity of PDL-MSCs [26]. In addition, a previous study demonstrated that sonic hedgehog signaling acted as a mediator of mechanical stress and involved in PDL-MSC proliferation in an autocrine manner [27].

On the other hand, the self-renewal capacity of PDL-MSCs decreased while donor aging. After 14 days of culture, PDL-MSCs from 15 donors with a mean age of 22.3 ± 7.7 years reached 97.6 ± 2.2% confluence; however, PDL-MSCs from 13 donors aged 62.6 ± 6.8 years showed only 42.8 ± 12.5% confluence [28]. Stem cell senescence has been known to be regulated by several factors, such as DNA damage, extrinsic force, and environmental changes of supporting tissues [29]. In addition, a previous study demonstrated the shortening of telomere and the activation of p53 and p16^INK4a^ declined highly proliferative and regenerative function of MSCs [30] (Figure 3(b)).

### 1.3. Stem Cell-Related Marker Expression of PDL-MSCs

Seo et al. firstly identified PDL-MSCs that expressed the cell surface molecules STRO-1 and CD146/MUC18, two early MSC-related markers present on bone marrow stromal cells and dental pulp stem cells [5]. PDL-MSCs also expressed CD44, CD90 (markers associated with stromal cells), CD105, and CD166 (markers associated with stromal cells and endothelial cells) [31]. In addition, Trubiani et al. showed a high expression of MSC-related markers such as CD10, CD26, CD29, CD73, and CD349/FZD9 in PDL-MSCs [32]. Interestingly, they also exhibited a strong expression of NANOG and OCT4, two critical transcription factors directing self-renewal and pluripotency of ES cells, in PDL-MSCs. Consistent with this result, Kawanabe et al. identified ES cell-related antigens including SSEA-1, SSEA-3, SSEA-4, TRA-1-60, TRA-1-81, ALP, SOX2, and REX1 in PDL-MSCs [33]. In contrast, PDL-MSCs lack expression of haematopoietic markers, CD34 (primitive haematopoietic progenitor marker), CD45 (pan-leucocyte marker), CD14 (monocyte/macrophage marker), and CD19 (B cell marker) [34]. Additionally, they were negative for CD40, CD80, and CD86 (markers associated with hematopoietic cells) [31]. The absence of these hematopoietic markers is known to be essential for defining mesenchymal cells. However, the specific markers for the identification of PDL-MSCs have not been discovered and the lack of a PDL-MSC-specific marker limits their precise isolation and characterization.

### 1.4. Immunomodulatory Effects of PDL-MSC

**Figure 4:**
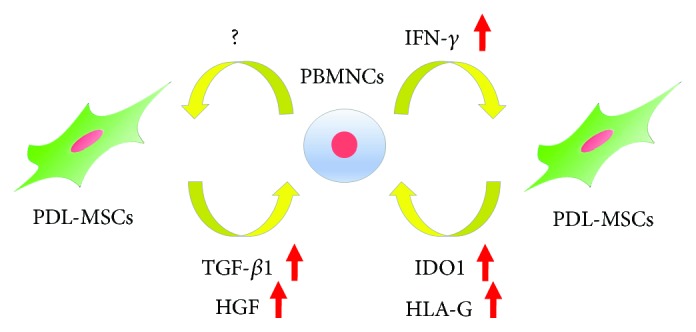
Schema of immunomodulatory effects of PDL-MSCs for PBMNCs. PBMNCs: peripheral blood mononuclear cells; TGF-*β*1: transforming growth factor-beta 1; HGF: hepatocyte growth factor; IFN-*γ*: interferon gamma; IDO-1: indoleamine 2,3-dioxygenase-1; HLA-G: human leukocyte antigen-G.

Immunomodulatory functions of MSCs have been reported in many different cell types including dental tissues such as PDL, dental pulp, root apical papilla, and gingiva [35, 36]. PDL-MSCs exhibited no expression of immune costimulatory factors such as CD40, CD80, CD86, and major histocompatibility complex class II antigen. These cells inhibited proliferation of peripheral blood mononuclear cells (PBMNCs) via cell cycle inhibition rather than that of apoptosis [31]. In addition, activated human PBMNCs induced PDL-MSCs to secrete some soluble factors and these factors partly inhibited proliferation of PBMNCs [31]. Interferon-gamma (IFN-*γ*), known to be secreted by activated PBMNCs, is an inflammatory cytokine involved in Th1 cell-mediated immune response. PDL-MSCs treated with IFN-*γ* exhibited the upregulation of indoleamine 2,3-dioxygenase-1 (IDO-1) expression and inhibited PBMNC proliferation (Figure 4) [31]. IL-12 is known to increase the expression of IFN-*γ* in various types of cells, and IL-12-treated PDL-MSCs showed a significant increase in IFN-*γ* expression as well as immunomodulatory factors such as IDO-1 and HLA-G [37]. These results suggested that PDL-MSCs exhibit their immunomodulatory properties through the IFN-*γ*-dependent pathway.

On the other hand, the IFN-*γ*-independent pathway was suggested to be involved in immunomodulatory functions of PDL-MSCs. TGF-*β*1 and HGF were considered as potential mediators of immunomodulatory effects in MSCs [38]. Wada et al. demonstrated their upregulation in PDL-MSCs cocultured with PBMNCs in the presence of an IFN-*γ*-neutralizing antibody [31] (Figure 4). IL-6, IL-8, and MCP-1 involved in immunomodulatory properties of PDL-MSCs and TLR antagonists rather than IFN-*γ* led to their production in PDL-MSCs [39]. Moreover, ERK signaling was suggested to regulate immunomodulatory activities of PDL-MSCs; LPS enhanced the inhibitory effect of PDL-MSCs for PBMNC migration and induced the expression of COX2 and IL-6 in PDL-MSCs; however, the ERK inhibitor significantly attenuated these effects and expressions [40].

Other reports discussed about immunomodulatory effects of PDL-MSCs on allogeneic T-cells. Shin et al. indicated that PDL-MSCs significantly decreased the level of nonclassical major histocompatibility complex glycoprotein CD1b via dendritic cells, resulting in defective T-cell proliferation [41]. MSC-like populations, derived from iPS cells generated from PDL cells, demonstrated the ability of suppression of T-cell effector cells, Th1/Th2/Th17 populations, and increased levels of Treg cells [42]. In an *in vivo* study, an allogeneic PDL-MSC sheet was used to cure periodontitis in a miniature pig periodontitis model and significant periodontal tissue regeneration was achieved in the allogeneic PDL-MSC transplantation group the same as that in the autologous group. This model exhibited that PDL-MSCs had low immunogenicity and marked immunosuppression through prostaglandin E2-mediated T-cell anergy [43]. Additionally, inflamed PDL-MSCs decreased their immunomodulatory properties compared with healthy PDL-MSCs [44]; inflamed PDL-MSCs indicated the diminished inhibition of T-cell proliferation and that PBMNCs cocultured with inflamed PDL-MSCs showed significantly less induction of CD4^+^CD25^+^FOXP3^+^ regulatory T-cells and IL-10 secretion. Furthermore, suppression of Th17 differentiation and IL-17 production were decreased in inflamed PDL-MSCs.

### 1.5. Regeneration of PDL Tissues Using PDL-MSCs

In 2004, PDL-MSCs were firstly transplanted into the animal models and their potential to reconstitute cementum/PDL-like structures became apparent [5]. This study strongly suggested the important roles of PDL-MSCs for the regeneration of the periodontium. Subsequently, many researchers have tied to regenerate the periodontium by the combination of PDL-MSCs, scaffolds, and growth factors. Ninomiya et al. transplanted rat PDL-MSCs seeded on hydroxyapatite (HA) disks under the fascia of the dorsal muscles of rat and demonstrated the formation of new bone-like tissues around HA disks [45]. HA and *β*-tricalcium phosphate (HA/TCP) were developed as bioceramics in the early 1980s and are the most common calcium phosphates applied for medical fields now. Therefore, several studies mixed human PDL-MSCs with HA/TCP and transplanted them into immunodeficient mice. They showed the successful formation of PDL-, bone-, and cementum-like tissues around the HA/TCP scaffold [46, 47]. Interestingly, a report demonstrated the formation of Sharpey's fiber-like tissue between PDL and cementum under similar conditions, indicating the complete regeneration of PDL tissues [48]. Jin et al. seeded recombinant human plasminogen activator inhibitor-1-treated human PDL-MSCs on the scaffolds constructed with HA/TCP and human tooth root dentin matrix and transplanted them into the dorsal surface of immunocompromised mice [49]. After 10 weeks, cementum-like structures were formed on the surface of the dentin matrix and PDL-like structures were generated outside of cementum-like structures. Stem cell sheets hold great promise in engineering three-dimensional biological tissues. Several studies tried to regenerate PDL tissues using human PDL-MSC cell sheets with several scaffolds and growth factors. Following the transplantation of HA/TCP scaffolds wrapped with human PDL-MSC cell sheets into the dorsal surface of immunodeficient mice, they formed bone-like structures [50]. The horse bone materials wrapped with human PDL-MSC cell sheets successfully generated cementum- and PDL-like structures when they were transplanted into the dorsal surface of immunodeficient rats [28]. Human PDL-MSC cell sheets were mixed with platelet-rich fibrin and then were sandwiched between HA/TCP and the human-treated dentin matrix to form PDL-like constructs. They successfully formed PDL, cementum, and blood vessel-like tissues after the transplantation into the immunodeficient mice [51].

To investigate the regenerative potential of PDL-MSCs and scaffolds in detail, they have been transplanted into animal models with PDL tissue defects. PDL-MSC cell sheets were transplanted into one-wall intrabony defects created on the mandibular teeth of dog with *β*-tricalcium phosphate/type I collagen scaffolds. After 8 weeks, PDL-MSCs induced the formation of new cementum, collagen fibers, and nerve fibers around the root surface [52]. In addition, rat PDL-MSCs were transplanted into artificially fabricated periodontal fenestration defects in rats with gelatin sponge scaffolds. Only 3 weeks later, the fenestration defects were filled with newly formed bone and cementum/PDL-like structures [53]. These results suggested that PDL-MSCs have the potential to form bone-, cementum-, and PDL-like tissues not only in the subcutis of the dorsal surface but also in the defects of the periodontium.

Furthermore, several factors have been suggested to affect the regenerative potential of PDL-MSCs.

A previous study demonstrated the induction of dysfunction in MSCs by inflammation [54]. Consistent with this report, PDL-MSCs obtained from the patients with periodontitis showed significantly lower bone formation than the cells acquired from the patients with healthy PDL tissues when they were transplanted into the subcutis of the dorsal surface in immunodeficient mice [55]. In addition, Gao et al. divided PDL-MSCs according to age of donors and compared their capability of PDL tissue regeneration using *in vivo* transplantation models. They demonstrated that PDL-MSCs derived from young donors revealed greater cementum- and PDL-like tissue formation than those from aged donors, suggesting that the senescence would exert an influence on the regenerative potential of PDL-MSCs *in vivo* [50].

### 1.6. Establishment of Human Periodontal Ligament Stem/Progenitor Cell Lines

The stem cell population in PDL tissue is quite rare; therefore, acquiring enough number of stem cells for the convenience and consistency of analyses is very difficult. Several researches tried to develop immortalized PDL stem cell lines using SV40 large T-antigen, human telomerase reverse transcriptase, human papillomavirus 16-related E6E7, Bmi-1, and BMP4. Consequently, immortalized PDL cell lines were established from mice, swine, and human cells [56, 57]. After that, Shirai et al. reported the establishment of clonal PDL cell lines that showed the potential to form mineralized nodules and vascular tube-like structures from swine immortalized PDL cell lines [58].

**Figure 5:**
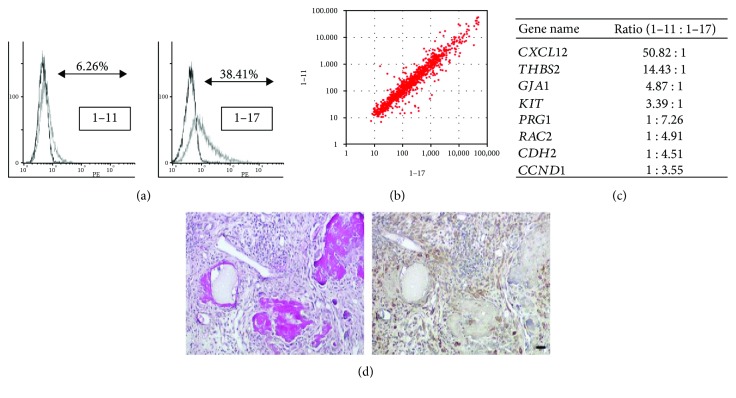
Comparison of MSC-related features between cell lines 1–11 and 1–17. (a) Flow cytometry analysis of cell lines 1–11 and 1–17 using antibodies reactive to p75NTR. (b) Scatterplot analysis of stem cell-related gene expression comparing cell lines 1–11 and 1–17. (c) The ratio of stem cell-related gene expression between cell lines 1–11 and 1–17. (d) HE (left) and human-specific vimentin staining of cell line 1–17 transplanted into immunodeficient mice. Red colors in the left panel show newly formed bone-like tissues. Vimentin-positive cells in the right panel mean the presence of transplanted cell line 1–17. Bar = 20 *μ*m.

Previously, our group succeeded to establish immortalized PDL cell lines using SV40 large T-antigen and human telomerase reverse transcriptase [59]. Then, two clonal human PDL cell lines with multipotency were isolated via limiting dilution. They were termed as cell lines 1–11 and 1–17. Both lines strongly expressed several MSC-related cell surface markers; however, their characteristic was suggested to be different from BM-MSCs; cell lines 1–11 and 1–17 were positive for PDL cell-related markers periostin and scleraxis, whereas bone marrow-derived MSCs were negative for them [60]. Interestingly, cell lines 1–11 and 1–17 revealed several different properties even though they were derived from the same PDL cell population. Cell line 1–11 had the potential to differentiate into osteoblasts and adipocytes [61], and cell line 1–17 could differentiate into osteoblasts, chondrocytes, adipocytes, and neural cells [60]. Cell line 1–17 strongly expressed ES cell-related markers *OCT4* and *NANOG* mRNA, whereas cell line 1–11 weakly expressed them [62]. Cell line 1–17 included more neural crest-related marker p75NTR-positive cells (38.41%) than cell line 1–11 (6.26%) (Figure 5(a)) and highly expressed neural crest-related marker genes, *p75NTR*, *SLAG*, *SOX10*, *NESTIN*, and *CD49D* [63]. Microarray analysis demonstrated that many stem cell-related genes were equivalently expressed in cell lines 1–11 and 1–17 (Figure 5(b)). On the other hand, they expressed several genes at different levels; cell line 1–11 showed the high expression of *CXCL12*, *GJA1*, *THBS2*, and *KIT* and cell line 1–17 highly expressed *RAC2*, *CDH2*, *PRG1*, and *CCND1* (Figure 5(c)).

**Figure 6:**
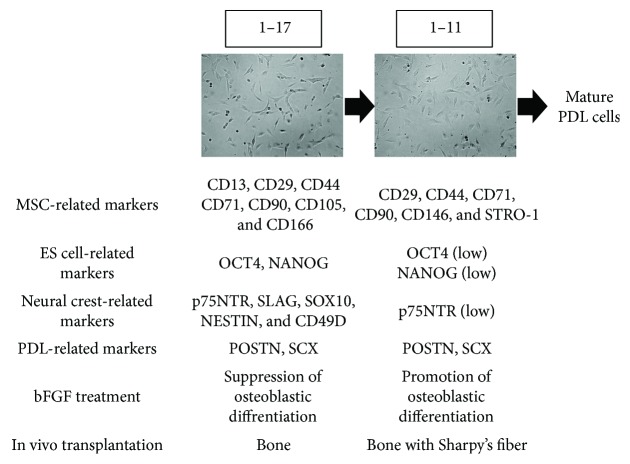
Schema of the different characteristics of cell lines 1–11 and 1–17. ES cells: embryonic stem cells; PDL: periodontal ligament; bFGF: basic fibroblast growth factor.

bFGF was reported to show the biphasic effect for osteoblastic differentiation; it suppressed calcified deposit formation in immature osteoblasts but promoted its formation in mature ones. After the culture of cell lines 1–11 and 1–17 with bFGF, calcified deposit production was enhanced in cell line 1–11, whereas it was suppressed in cell line 1–17 [60]. Following the subcutaneous transplantation of both lines into the dorsal side of immunodeficient mice with *β*-TCP, cell line 1–11 produced bone-like tissues including Sharpey's fiber-like tissues [61]. Cell line 1–17 also revealed the potential to generate bone-like tissues; however, Sharpey's fiber-like tissues were not detected in them (Figure 5(d)). Following the injection of both lines into artificially fabricated periodontal defects, cell line 1–11 was located on the surfaces of alveolar bone and tooth root and in the PDL tissue; however, cell line 1–17 was observed only in the PDL tissue [62]. These results suggest the typical characteristics of stem cells in cell lines 1–11 and 1–17 and their difference in maturity. Basically, cell line 1–17 would be at a much earlier stage of differentiation than cell line 1–11 according to the differences of multipotency, ESC- and neural crest-related marker expression, the response to bFGF in osteoblastic differentiation, and the results of transplantation assays (Figure 6).

### 1.7. Future Prospective

PDL-MSCs are promising cells; however, their rarity prevents their application for use in studies of PDL regeneration. In addition, elderly people need PDL regeneration than young people do because the prevalence and severity of periodontal disease are increasing with age. However, the multipotent and self-renewal capacity of PDL-MSCs decreased while donor aging as described above. Therefore, easily obtaining large numbers of PDL from elderly people is significantly important to achieving the development of PDL regenerative therapy. iPS cells have attracted a great deal of attention because of their generation from somatic cells and high multidifferentiation and self-renewal potential [8]. Interestingly, undifferentiated iPS cells formed mature teratoma tissues; however, iPS cell-derived differentiated cells exerted no teratoma formation [64]. Based on these results, we tried to establish PDL-MSC-like cells from human iPSCs. Our recent study demonstrated the generation of human iPS cell-derived PDL-MSCs that expressed both stem cell- and PDL cell-related markers using ECM derived from PDL cells [65]. Human iPS cell-derived PDL-MSCs would overcome the rarity of PDL-MSCs and suppress the risk of iPS cell-related tumorigenesis; therefore, these cells hold promise to realizing PDL tissue engineering. Further work is now essential to confirm their safety and to evaluate their potential to regenerate PDL tissues *in vivo*.

## 2. Conclusion

As reviewed in this article, PDL-MSCs show MSC properties including multipotency, self-renewal capacity, MSC-related marker expression, and immunomodulatory effects. In addition, they perform crucial roles in the regeneration of PDL tissue. Therefore, PDL-MSCs will provide novel approaches to periodontal disease and give a great promise for new therapy in destructed PDL tissues.
